# Assessment of the Prevalence of the Use of Nasal Decongestants Among the General Population in Saudi Arabia

**DOI:** 10.7759/cureus.31892

**Published:** 2022-11-25

**Authors:** Ahmad S Alharthi, Saud A Alharthi, Abdulaziz F Altowairqi, Shrooq H Alswat, Marwan F Alnofaie

**Affiliations:** 1 Otolaryngology - Head and Neck Surgery, Alhada Hospital for Armed Forces, Taif, SAU; 2 Otorhinolaryngology, King Faisal Medical Complex, Taif, SAU; 3 Otorhinolaryngology - Head and Neck Surgery, King Abdulaziz Specialist Hospital, Taif, SAU; 4 General Medicine, King Faisal Medical Complex, Taif, SAU

**Keywords:** saudi arabia, population, pattern of use, prevalence, utilization, nasal congestion, nasal decongestants

## Abstract

Background

A topical nasal decongestant (NDC) is widely prescribed in ENT practice and used as self-medication because it is available over the counter, which makes it an easily accessible medication. Due to its common and long-term use, it is associated with serious nasal complications. It is commonly self-administrated in many otolaryngology diseases like the common cold, sinusitis, and acute or chronic rhinitis. The long-term usage of nasal decongestants is associated with significantly increased side effects.

Aim

To assess the prevalence of the usage of nasal decongestants among the general population in Saudi Arabia ad the pattern of its use.

Methodology

A questionnaire-based, cross-sectional survey was applied to level all available populations in Saudi Arabia. Participants with ages aged 10 to 60 years old in Saudi Arabia were invited to participate in the survey. Data were collected from participants using a predesigned online questionnaire. The questionnaire included the participant's demographic data, NDC use, and pattern of use. The questionnaire was uploaded online by researchers and their friends using social media platforms.

Results

A total of 1456 participants completed the study questionnaire. Participants ages ranged from 10 to 60 years with a mean age of 26.9 ± 12.4 years old. Exact 585 (40.2%) participants were males and 1270 (87.2%) were from urban regions. A total of 657 (45.1%) respondents reported using nasal decongestants while 799 (54.9%) did not use NDC. As for the duration of use, 70.8% used NDC for less than five days and 13.5% used it for 5-15 days. The most reported causes of using NDC were nasal obstruction (62.7%) and common cold (25.7%).

Conclusions

In conclusion, the study revealed that the frequency of using nasal decongestants was common (45.1%) in the study. More efforts should be paid to improve public awareness regarding indications, duration of use, and method of using nasal decongestants to avoid rebound reactions that may affect patients’ daily life activities.

## Introduction

Nasal congestion mainly occurs due to the dilatation of nasal blood vessels that enlarge to partially or even completely obstruct the airflow in one both nasal passages. Nasal obstruction with nasal congestion can be discriminated from anatomical obstruction through the application of a topical nasal decongestant spray [1]. The topical nasal decongestant constricts nasal blood vessels and opens up the airways. Nasal decongestants (NDC) are medications usually used in otolaryngology for the management of nasal and sinus congestion, seasonal rhinitis, common cold, and allergic rhinitis [2]. Allergic rhinitis is a nasal disorder that is diagnosed among about 10% to 20% of the population and 15% to 25% of children and young adults [3]. Nasal decongestants are strong vasoconstrictive agents and are usually used in relieving nasal congestion associated with some nasal conditions, including allergic rhinitis, upper respiratory allergies, rhinosinusitis, nasal polyps, and hypertrophy [4,5]. Nasal decongestants feature rapid onset effects with the fast relief of symptoms. Some decongestants, such as brimonidine tartrate, are sometimes prescribed for ocular mucosa to treat other conditions like conjunctivitis, ocular hypertension, and open-angle glaucoma [6]. Though the use of nasal decongestants for a long duration may cause adverse effects such as iatrogenic rhinitis [7]. Consequently, the local application of decongestants may be used only for short periods of time and not recommended to be used for a period exceeding four or five days, to avoid the hazard of mucosal injury and rebound vasodilation [8]. Besides the prolonged use of decongestants affects the sensitivity of alpha receptors, with a need for higher doses in shorter pauses to get the same effect. So patients should use excessive doses of nasal decongestants [9]. Many drugs are used for nasal congestion but the most effective drugs used comprise phenylephrine, pseudoephedrine, oxymetazoline, naphazoline, and xylometazoline [10,11]. Mostly having antihistamines with decongestants showed significant relief in allergic conditions [12]. It is thought that the use of nasal decongestants is common in patients who have nasal symptoms, especially patients with nasal obstruction and it has serious effects on health but there is still a lack of studies to identify the size of the problem is Saudi Arabic and more specifically in Taif city. The main aim of this study was to identify the prevalence of using nasal decongestants among the general population in Saudi Arabia and their pattern of use.

## Materials and methods

A questionnaire-based cross-sectional survey was applied to all available populations in Saudi Arabia. Participants aged 10 to 60 years old in Saudi Arabia were invited to participate in the survey. Data were collected from participants using a pre-designed online questionnaire. The study authors constructed the questionnaire after a comprehensive literature review and expert consultation. The tool was reviewed by a group of three experts in the study issue to check its clarity and content validity. The questionnaire included the following data: participants' socio-demographic data like age, gender, education, and monthly income. Also, participants’ habits, including smoking, work section, and their awareness regarding nasal decongestant use patterns were included. The second section covered the frequency of nasal decongestant use frequency and patterns such as duration of use, frequency of daily use, type of used NDC, improvement after use, and related complications. The questionnaire was uploaded online using social media platforms by researchers and their friends and all eligible persons were invited to fill it out after explaining the purpose and confirming their data confidentiality.

The IRB Registration Number for Research was KACST, KSA, HAP-02-T-067; approval number 589.

After data were extracted, it was revised, coded, and fed to statistical software IBM SPSS version 22 (IBM Corp, Armonk, NY). All statistical analysis was done using two-tailed tests. A p-value less than 0.05 was statistically significant. Descriptive analysis based on frequency and percent distribution was done for all variables, including participants’ socio-demographic data related to gender, residence, education, marital status, and income. Also, smoking history was tabulated with work area and perception of using nasal decongestants. The prevalence of use of nasal decongestants was graphed while the pattern of use was presented in frequency tables. Crosstabulation was used to assess factors associated with nasal decongestant use among study participants and to assess factors associated with complications experienced. Relations were tested using the Pearson chi-square test and the exact probability test for small frequency distributions.

## Results

Table 1 shows the bio-demographic data of the study participants in Saudi Arabia. A total of 1456 participants completed the study questionnaire. Exactly 67.3% were from the Western region, 13.5% were from the Eastern region, 8.4% from the Central region, 8.2% from the Southern region, and 2.5% were from the Northern region. The participant's ages ranged from 10 to 60 years with a mean age of 26.9 ± 12.4 years. Exactly 585 (40.2%) participants were males and 1270 (87.2%) were from urban regions. Considering marital status, 785 (53.9%) were married and 610 (41.9%) were single. As for educational level, 1120 (76.9%) were university graduates while 293 (20.1%) had a secondary level of education. Monthly income less than 5000 SR was reported among 34.1% of the participants while 43.5% had a monthly income of 5000-15000 SR. A total of 182 (12.5%) were smokers and 1195 (82.1%) were non-smokers. Among smokers, 129 (70.5%) smoked one to two packets per day. As for job titles, 435 (29.9%) were not working/students, 621 (42.7%) worked in the non-healthcare sector, and 400 (27.5%) worked in the healthcare sector. Exactly 1243 (85.4%) participants thought there are negative effects of using decongestants for a long time, and 864 (59.3%) reported the recommended duration for using decongestants of three to five days while 331 (22.7%) reported less than 3 days.

**Table 1:** Bio-demographic data of study participants in Saudi Arabia SR: Saudi Riyal

Bio-demographic data		No	%
Region	Central	123	8.4%
	Northern	36	2.5%
	Eastern	197	13.5%
	Western	980	67.3%
	Southern	120	8.2%
Age in years	10-18	41	2.8%
	19-30	722	49.6%
	31-40	315	21.6%
	41-60	378	26.0%
Gender	Male	585	40.2%
	Female	871	59.8%
Residence	Urban	1270	87.2%
	Rural	186	12.8%
Marital status	Single	610	41.9%
	Married	785	53.9%
	Divorced / widow	61	4.2%
Educational level	Below secondary	43	3.0%
	Secondary	293	20.1%
	University / above	1120	76.9%
Monthly income	< 5000 SR	496	34.1%
	5000-15000 SR	633	43.5%
	> 15000 SR	327	22.5%
Smoking	Current smoker	182	12.5%
	Ex-smoker	79	5.4%
	Non-smoker	1195	82.1%
How many packets / day	< 1 packet / day	47	25.7%
	1-2 packets / day	129	70.5%
	> 2 packets / day	7	3.8%
Work section	Not working / student	435	29.9%
	Healthcare sector	400	27.5%
	Non-healthcare sector	621	42.7%
Do you think there are negative effects of using decongestants for a long time?	Yes	1243	85.4%
	No	213	14.6%
Recommended duration for using decongestants	Only 1 day	88	6.0%
	< 3 days	331	22.7%
	3-5 days	864	59.3%
	No recommended duration	173	11.9%

**Figure 1:**
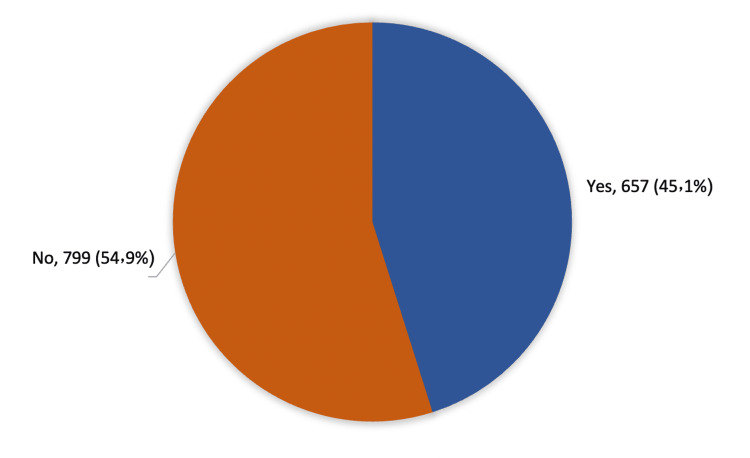
Prevalence of using nasal decongestants among the general population in Saudi Arabia

Figure 1 shows that a total of 657 (45.1%) respondents reported using nasal decongestants while 799 (54.9%) did not.

Table 2 shows the pattern of nasal decongestant use in the study population in Saudi Arabia. As for the duration of use, 70.8% used NDC for less than 5 days, and 13.5% used it for 5-15 days. Exactly 41.1% of the users reported using NDC 2 times/day while 22.8% used it 3 times/day. The most used NDC were Otrivin (76%), followed by Xylomet (9.4%), Aphist (6.2%), and Decozal (2.1%). The most reported causes of using NDC were nasal obstruction (62.7%), common cold (25.7%), itchiness (3.7%), and sneezing (3.5%). A total of 26% of the users reported using oral antihistamines with NDC, 22.1% used paracetamol, and 43.5% not used any other medications. NDC use was recommended by physicians among 54.8% of the users, by pharmacists among 21%, 12.8% by family, and 4.3% by friends. Exactly 65.3% were advised about how to use NDC. A total of 89.6% of the users consume one bottle of NDC per month, and 95.7% reported improved symptoms with NDC use while only 8.1% were diagnosed by a physician for any complications.

**Table 2:** Pattern of nasal decongestant use among the study population in Saudi Arabia NDC: nasal decongestant

Pattern of use		No	%
Duration of using decongestant	< 5 days	465	70.8%
	5-15 days	89	13.5%
	15-30 days	24	3.7%
	2-6 months	14	2.1%
	7-12 months	16	2.4%
	2-5 years	16	2.4%
	> 5 years	33	5.0%
Frequency of using decongestants / day	1 time / day	120	18.3%
	2 times / day	270	41.1%
	3 times / day	150	22.8%
	4 times / day	14	2.1%
	5 or more / day	14	2.1%
	Only with symptoms	89	13.5%
Type of used nasal decongestant	Otrivin	499	76.0%
	Xylomet	62	9.4%
	Apihist	41	6.2%
	Decozal	14	2.1%
	Nasonex	9	1.4%
	Avamys	6	.9%
	Xylo-Acino	6	.9%
	Otrizethic	3	.5%
	Rhinocort	3	.5%
	Saltwater	3	.5%
	Levocapastine	2	.3%
	Rinoclenil	2	.3%
	Livostin	1	.2%
	Nasacort	1	.2%
	Nasonex	1	.2%
	RHINASE	1	.2%
	Rinoclenil100	1	.2%
	Sinoclear	1	.2%
	Sterimar	1	.2%
Causes of using nasal decongestants	Nasal obstruction	412	62.7%
	Common cold	169	25.7%
	Itchiness	24	3.7%
	Sneezing	23	3.5%
	Rhinosinusitis	16	2.4%
	Allergic rhinitis	13	2.0%
Other medications used with NDC	None	286	43.5%
	Oral antihistamine	171	26.0%
	Paracetamol	145	22.1%
	Others	55	8.4%
NDCs were recommended by	Physician	360	54.8%
	Pharmacist	138	21.0%
	Family	84	12.8%
	Friends	28	4.3%
	My self	25	3.8%
	Internet	22	3.3%
Any person offered advice on how to use a nasal decongestant	Yes	429	65.3%
	No	228	34.7%
Main site for purchasing the nasal decongestant	Pharmacy	470	71.5%
	Hospital	187	28.5%
How many bottles used per month?	One	589	89.6%
	Two	45	6.8%
	Three	9	1.4%
	4 / more	14	2.1%
Do your symptoms improve with the use of a decongestant?	Yes	629	95.7%
	No	28	4.3%
Are you diagnosed by a physician for any complications?	Yes	53	8.1%
	No	604	91.9%

Table 3 shows factors associated with nasal decongestant use among study participants in Saudi Arabia. The highest reported utilization rate was among young participants (63.4% of those aged 10-18) compared to 43.8% of those aged and 51.9% for those aged 41-60 years with recorded statistical significance (P=.001). Also, 61.6% of single participants used NDC versus 49.2% of the separated group (P=.001). Additionally, NDC use was reported among 60.3% of healthcare workers in comparison to 50.4% of non-healthcare workers (P=.007). Besides, 56.6% of those who think there are negative effects of using decongestants for a long time used NDC versus 45.1% of others who did not (P=.002).

**Table 3:** Factors associated with nasal decongestant use among study participants in Saudi Arabia P: Pearson's X2 test; $: Exact probability test; * P < 0.05 (significant); SR: Saudi Riyal

Factors		Do you use a nasal decongestant?				p-value
		Yes		No		
		No	%	No	%	
Region	Central	53	43.1%	70	56.9%	.638
	Northern	18	50.0%	18	50.0%	
	Eastern	95	48.2%	102	51.8%	
	Western	432	44.1%	548	55.9%	
	Southern	59	49.2%	61	50.8%	
Age in years	10-18	15	36.6%	26	63.4%	.001*^$^
	19-30	287	39.8%	435	60.2%	
	31-40	172	54.6%	143	51.6%	
	41-60	183	48.4%	195	51.9%	
Gender	Male	262	44.8%	323	55.2%	.832
	Female	395	45.4%	476	54.6%	
Residence	Urban	575	45.3%	695	54.7%	.761
	Rural	82	44.1%	104	55.9%	
Marital status	Single	234	38.4%	376	61.6%	.001*
	Married	392	49.9%	393	50.1%	
	Divorced / widow	31	50.8%	30	49.2%	
Educational level	Below secondary	18	41.9%	25	58.1%	.891
	Secondary	131	44.7%	162	55.3%	
	University / above	508	45.4%	612	54.6%	
Monthly income	< 5000 SR	212	42.7%	284	57.3%	.096
	5000-15000 SR	306	48.3%	327	51.7%	
	> 15000 SR	139	42.5%	188	57.5%	
Smoking	Current smoker	95	52.2%	87	47.8%	.122
	Ex-smoker	35	44.3%	44	55.7%	
	Non-smoker	527	44.1%	668	55.9%	
Work section	Not working / student	190	43.7%	245	56.3%	.007*
	Healthcare sector	159	39.8%	241	60.3%	
	Non-healthcare sector	308	49.6%	313	50.4%	
Do you think there are negative effects of using decongestants for a long time?	Yes	540	43.4%	703	56.6%	.002*
	No	117	54.9%	96	45.1%	

Table 4 shows the factors associated with the side effects/complications of nasal decongestants among study participants in Saudi Arabia. Complications were reported among all cases who had NDC by themselves without a prescription versus 91.4% of others who used it through physician advice (P=.049). Also, 93.3% of those who consume two bottles of NDC per month developed complications compared to 55.6% of those who used three bottles (P=.001).

**Table 4:** Factors associated with the side effects/complications of nasal decongestant among study participants in Saudi Arabia P: Pearson X2 test; $: Exact probability test; * P < 0.05 (significant); NDC: nasal decongestant

NDC use	Are you diagnosed with any complications by a physician?				p-value
	Yes		No		
	No	%	No	%	
Duration of using decongestant					.149
< 5 days	30	6.5%	435	93.5%	
5-15 days	11	12.4%	78	87.6%	
15-30 days	1	4.2%	23	95.8%	
2-6 months	1	7.1%	13	92.9%	
7-12 months	3	18.8%	13	81.3%	
2-5 years	2	12.5%	14	87.5%	
> 5 years	5	15.2%	28	84.8%	
Frequency of using decongestants / day					.161
1 time / day	12	10.0%	108	90.0%	
2 times / day	17	6.3%	253	93.7%	
3 times / day	9	6.0%	141	94.0%	
4 times / day	3	21.4%	11	78.6%	
5 or more / day	2	14.3%	12	85.7%	
Only with symptoms	10	11.2%	79	88.8%	
NDC was recommended by					.048*^$^
Physician	31	8.6%	329	91.4%	
Pharmacist	9	6.5%	129	93.5%	
Family	6	7.1%	78	92.9%	
Friends	2	7.1%	26	92.9%	
My self	0	0.0%	25	100.0%	
Internet	5	22.7%	17	77.3%	
How many bottles are used per month?					.001*^$^
One	43	7.3%	546	92.7%	
Two	3	6.7%	42	93.3%	
Three	4	44.4%	5	55.6%	
4 / more	3	21.4%	11	78.6%	

## Discussion

Nasal congestion use patterns and epidemiology are not properly studied in the general population and the majority of congestion-related studies were among patients with diagnosed rhinologic disorders. The most reported cause of nasal congestion is allergic rhinitis [13,14]. Globally, the prevalence of allergic rhinitis-related congestion showed an upward trend [15,16]. Nasal decongestant drugs are extensively used in rhinology with reported practice among the general population. Topical nasal decongestants are available as over-the-counter drugs. Nasal decongestant medications, including alpha-adrenergic agonists like oxymetazoline, xylometazoline, phenylephrine hydrochloride, pseudoephedrine, naphazoline hydrochloride, tetrahydrozoline hydrochloride, clomazone, tramazoline, hydroxy amphetamine, tuaminoheptane, and phenylpropanolamine, are sympathomimetic factors that emulate sympathetic central nervous system activity in the body [17,18].

The current study aimed to assess the frequency of using nasal decongestants among the general population in Saudi Arabia and to identify the pattern of use and the factors influencing the long-term use of nasal decongestants. The majority of the participants (more than three-quarters) were non-smokers while about three-quarters of the smokers used one to two packs daily. Also, the study revealed that more than three-quarters (85%) of the study participants thought there are negative effects of using decongestants for a long time, and more than half of them (59%) reported that the recommended duration for using decongestants is three to five days. Topical nasal decongestants recommended that the duration of use should not exceed five days [17], as rebound congestion is the common side effect reported due to the misuse of nasal decongestant medications, particularly in their topical agents [19]. Globally, some countries recommended the use of decongestion drugs is limited to a maximum of 10 days because of the risk of developing rhinitis medicamentosa [20]. 

Regarding the prevalence of using nasal decongestants among the general population in Saudi Arabia, the study showed that less than half of the participants (45.1%) reported using nasal decongestants. It was used for less than five days among about 70% of participants and less than half of them used it two times daily. About three-quarters of the users reported using Otrivin (local decongestant), but much fewer cases used Xylomet and Apihist. Nasal obstruction was the main cause of using NDC (among two-thirds of the users), where one-fourth of them used NDC for the common cold, and this explains why topical agents were most used. The physician was the main prescriber for using NDC (among more than half of the users) while one-fifth of the cases were advised by pharmacists. The study also showed that two-thirds of the users received advice on how to use NDC and the vast majority of them experienced an improvement in the symptoms, whereas less than 10% had complications due to NDC use. Alyahya KA et al. reported that 68.5% of Saudi adults had used nasal decongestants, whereas 66.5% had used them with a prescription and 31.5% without a prescription [21]. Yoo JK et al. found that four (40%) volunteers had a history of allergic rhinitis and 2 (20%) used topical nasal steroids [18]. Aljibori AS assessed 300 patients (150 male,150 female) with full history-taking and complete nasal examination [22]. About 13.3% were complaining of rhinitis medicamentosa. A total of five patients (12.5%) used nasal decongestants for two weeks, 15 patients (37.5%) used the medications for four weeks, and 20 patients (50%) used them for more than six weeks. Lenz D et al. reported that about 71.8% of university students had already used topic nasal decongestants, 64.3% had used this medication for at least 15 days, and 64.6% had started using it due to nasal obstruction [17]. Also, the pharmacist was the main health professional who provided patients with guidance on how to use this medication. Regarding the associated benefits and complications of NDC use, Taverner D conducted a systematic review and concluded a small but statistically significant reduction of 6% in subjective symptoms after a single dose of decongestant compared with a placebo [23]. Additionally, there was a decrease in nasal airway resistance. With repeated doses, nasal decongestants caused a very small statistical benefit of 4% over three to five days with a decrease in nasal airway resistance. Two studies revealed relatively few adverse events and only a small increased risk of insomnia with pseudoephedrine compared to placebo.

The strengths of our study include the large sample size, diversity, and national distribution of participation. Our limitations include the electronically distributed questionnaire.

## Conclusions

In conclusion, the study revealed that the frequency of using nasal decongestants was common (45.1%) in the participants. The main cause of use was nasal obstruction. Nasal decongestants were mainly used for less than five days, which is the most recommended duration to avoid adverse events. Health care staff (physicians and pharmacists) had a major role in advising the use of nasal decongestants with a high improvement rate in symptoms and low experienced side effects. More efforts should be paid to improve public awareness regarding indications, duration of use, and method of using nasal decongestants to avoid rebound reactions that may affect the patients’ daily life activities.
